# Dormant Polymers and Their Role in Living and Controlled Polymerizations; Influence on Polymer Chemistry, Particularly on the Ring Opening Polymerization

**DOI:** 10.3390/polym9120646

**Published:** 2017-11-25

**Authors:** Stanislaw Penczek, Julia Pretula, Piotr Lewiński

**Affiliations:** Centre of Molecular and Macromolecular Studies of Polish Academy of Sciences, Sienkiewicza 112, 90-363 Lodz, Poland; jpretula@cbmm.lodz.pl (J.P.); lewinski@cbmm.lodz.pl (P.L.)

**Keywords:** dormant polymers, living polymerization, controlled polymerization, ring-opening polymerization

## Abstract

Living polymerization discovered by Professor Szwarc is well known to all chemists. Some of the living polymerizations involve dormancy, a process in which there is an equilibrium (or at least exchange) between two types of living polymers, namely active at the given moment and dormant at this moment and becoming active in the process of activation. These processes are at least equally important although less known. This mini review is devoted to these particular living polymerizations, mostly polymerizations by the Ring-Opening Polymerization mechanisms (ROP) compared with some selected close to living vinyl polymerizations (the most spectacular is Atom Transfer Radical Polymerization (ATRP)) involving dormancy. Cationic polymerization of tetrahydrofuran was the first one, based on equilibrium between oxonium ions (active) and covalent (esters) dormant species, i.e., temporarily inactive, and is described in detail. The other systems discussed are polymerization of oxazolines and cyclic esters as well as controlled radical and cationic polymerizations of vinyl monomers.

## 1. Introduction

Professor Michael (Michał in Polish) Szwarc’s contributions to the chemical science are not only related to polymers and to his discovery of living and (less known) dormant polymers [1]. Dormant polymers, reversibly formed from the active ones, are at the basis of several living/controlled polymerizations described in the present paper.

Moreover, Professor Szwarc is also well known for his work on the bond energy (continued for some time in Syracuse) and on the electron transfer phenomena [2,3].

In 1956, during discussion with Samuel Weissman on the electron transfer in aromatic compounds, Szwarc asked Weissman about styrene (cit.) “Did you transfer electrons to styrene?” The answer he received was: (cit.) “No use, it polymerizes” [4]. Szwarc then realized that electron transfer in the case of styrene not only leads to polymerization, but also to creation of living active species which was indicated by the persistent red color. Since then, working with his students/coworkers, Moshe Levy, Ralph Milkovich, Joseph Jagur, Johannes Smid and several postdocs, he has answered all major questions concerning the anionic polymerization, and is acknowledged by all chemists to be the “father of the living polymerization”. Thus, despite his other important contributions to chemistry, the discovery of the living and dormant polymers is rightly considered to be his greatest achievement. In the recently published “Perspectives” by Grubbs and Grubbs [5], it is amply shown that discovery of the living polymerization revolutionized practically all areas of polymer chemistry and is still a driving force for novelty in general chemistry and material sciences [6].

The issue strongly connected with living-active polymers, yet not that well known by many chemists, are living-dormant polymers; both have been extensively described in the monumental Szwarc’s monograph on anionic polymerization [1]. The concept is based on the Szwarc’s observation that, in the anionic polymerization of styrene in the presence of anthracene, the temporarily active and inactive (dormant) species are formed reversibly [7].

It will be shown in the present mini review that dormant polymers play a decisive role in understanding living-controlled processes such as ROP [8] and are a fundamental aspect of the controlled radical polymerizations [9,10,11,12].

## 2. Basic Definitions and Their Importance for Better Understanding of the Processes under Study: “Living”, “Controlled”, and “Dormant”

The expressions listed in the Section 2 title are often used in contexts that are not completely proper. The definition given by International Union of Pure and Applied Chemistry (IUPAC), based on the Szwarc’s description of living and dormant polymers, is as follows: “Ideally, living polymers propagate while their termination or chain transfer are rigorously prevented” [4].

The expression “living polymers” was coined soon after the first experiments had been performed and was used in the two first papers that were published (not without difficulties, due to the expression “living” being restricted for biosciences). These famous papers appeared in 1956 in Nature and Journal of American Chemical Society [13,14].

It is not sufficiently appreciated in general chemistry, as this discovery is important not merely for polymer chemistry. Indeed, until Szwarc’s discovery, chain reactions were described (since Bodenstein’s discovery of chain reactions [15,16]) as composed of at least three elementary reactions: initiation, propagation and termination. This has been described in detail, for instance, by Nicolai Semenov and Sir Cyril Hinshelwood, Nobel Prize Laureates for their work on the chain reactions [17,18].

In contrast to these processes, in living polymerizations, the process is composed exclusively of initiation and propagation, eliminating termination. Therefore, it has led to the novel kind of steady state processes. Until the discovery of living polymers, steady state reactions (as described by Bodenstein [15]) resulted from an interplay of formation of chain carriers in the initiation reaction and their disappearance in the termination reaction by interaction of two chain carriers. This principle is at the bases of the kinetics of the large number of chain reactions, including free radical polymerizations.

The novel concept of the steady state is based on the invariant concentration of active centers (growing chains) equal from the very beginning (at certain conditions) to the concentration of the initiator of the polymerization. This approach is true when initiation is complete and the rate of initiation is faster than the rate of propagation. Such processes are considered as fully controlled. However, living polymerizations are not restricted to this particular instance. Slow initiation accompanying fast propagation may also provide living polymers although polymerization is not controlled.

These general phenomena are (or should be) taught in every course of polymer science and technology and are used by hundreds of authors in thousands of papers, yet, the actual limitations, discussed further in the text, are seldom taken into the account.

IUPAC has defined the terms “living”, “controlled” and “dormant”, simultaneously putting to rest several other expressions that have proven to be no long necessary and were often very confusing (e.g., “immortal”, “quasi living”, etc.) [19].

Therefore, the living polymerization is (cit.) “a chain polymerization from which irreversible chain transfer and chain termination are absent. In many cases, the rate of chain initiation is fast compared with the rate of chain propagation, so that the number of kinetic-chain carriers is essentially constant throughout the polymerization” [20].

The term “Controlled polymerization” indicates (cit.) “control of a certain kinetic feature of a polymerization or structural aspect of the polymer molecules formed, or both” [19]. Thus, for living polymerization, termination and irreversible chain transfer should be absent, although reversible deactivation (e.g., reversible transfer), leading to dormant macromolecules, is permitted, as is slow initiation. It may therefore happen that a living polymerization is not a controlled one (due to slow initiation) and controlled polymerizations may not be living, when molar mass and end-groups are under control only within a certain range of conversions and polymerization degrees (usually limited).

As the criterion of the absence of termination and irreversible transfer, Szwarc quoted [21] (pp. 14–15) an equation derived in our group [22]: ln(1 − *P*_n_·[*I*]_0_/[*M*]_0_) = −*k*_p_·[*I*]_0_·*t*, where *I*, *M* and *t* are the concentration of the initiator, the concentration of the monomer and time, respectively. *P*_n_ stands for the degree of the polymerization. The linearity of the plot of this equation indicates the simultaneous absence of these two mentioned reactions and is characteristic for both living and controlled polymerizations.

Dormant species are formed in reversible chain deactivation and this process is described by IUPAC as follows (cit.) “deactivation of a chain carrier in a chain polymerization, reversibly converting an active center into an inactive one and then, within the average lifetime of a growing macromolecule, regenerating an active center on the same original carrier, hence, the temporarily deactivated species created in this process are often described as dormant” [19]. The dormant polymers, i.e., macromolecules, are thus living and only temporarily become unable to grow.

Szwarc not only observed temporarily inactive species, coining an expression “dormant”, but also made the following statement on the living-dormant polystyrene complexed with anthracene [7] (cit.) “Complex is in equilibrium with its components, on addition of styrene the polymerization ensues. The exchange between the free living and those complexed with anthracene (dormant) is sufficiently rapid to distribute the polymerized monomer among all polymer molecules”. Thus, the importance of the fast exchange has clearly been stated as a necessary condition for low dispersity (*Ð*), as computed, for example, for the controlled radical polymerization (e.g., ATRP) by Matyjaszewski [23].

The term “immortal”, which is not supported by IUPAC, was introduced by Inoue for systems with reversible chain transfer (and formation of dormant species) [24]. It is still used by some authors, although IUPAC definition for “living” encompasses reversible transfers. Indeed, as already stated, the resulting from reversible transfer dormant macromolecules retain an ability to grow.

Definition of these terms has been important not only from the semantic point of view but also to allow researchers to look at their systems in a more careful manner and to elaborate such conditions for the studied processes that would conform them to these definitions, and, therefore, to better control of the studied systems.

## 3. Dormancy

As mentioned above, it had been in the school of Syracuse that important phenomenon of “dormant polymers” was first observed and defined. Later, understanding of this principle by several other researchers allowed conversion of the otherwise nonliving polymerizations into the living ones. This concept was introduced to account for the fact that “living ends can be protected from termination, but still be reversibly active for propagation”, as recently remained by Levy [6].

There is a plethora of reviews on living and controlled polymerization, yet dormancy has never been comprehensively reviewed. Probably the only exceptions are: a subsection titled “Living and Dormant Polymers” in the last book by Szwarc (published in 1996) [25] (p. 17) and the paper published by our group in 1995 under the title “Polymerizations with contribution of covalent and ionic species” [26].

The present mini review is merely giving a few examples of chain polymerizations, being living-dormant and controlled-dormant, particularly in the ROP and compared with vinyl polymerizations.

## 4. Dormant Species in the Ring-Opening Polymerization (ROP)

**Scheme 1:**
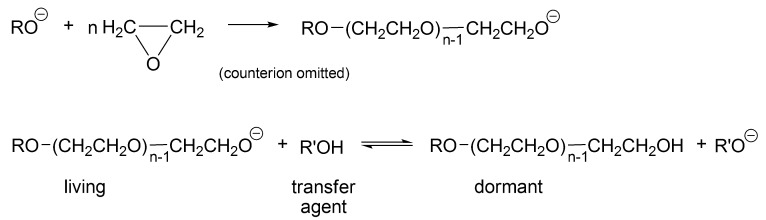
An anionic polymerization of ethylene oxide in the presence of an alcohol.

Probably, the first process that would be called today “living-dormant” was described by Flory already in 1944 for an anionic polymerization of ethylene oxide in the presence of an alcohol (Scheme 1) [27]. This process leads to the Poisson distribution of molar masses derived based on this polymerization [27,28].

As follows from Scheme 1, R’OH is the chain transfer agent that is present from the beginning of the polymerization (at first, as a low molar mass alcohol and later as macro alcohol). RO(CH_2_CH_2_O)CH_2_CH_2_OH is at the same time a chain transfer agent and a dormant, temporarily inactive polymer chain, reversibly converting in the reaction with macroanion R’O^−^ into an active form—a living macromolecule.

In several, later studied ring-opening polymerizations, living-dormant interconversions take place. However, species that look dormant may not necessarily be “completely inactive” and may retain partial activity as it has been documented in the polymerization of tetrahydrofuran (THF) initiated with triflic acid (trifluoromethane sulfonic acid, TfOH) [29], or in the polymerization of oxazolines initiated with alkylhalides [30]. In these systems, dormant macromolecules may have some activity yet many times less than the active ones and rightly could be considered as dormant.

**Scheme 2:**
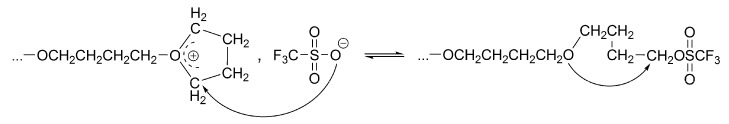
The active-inactive (dormant) interconversion in the polymerization of THF initiated with TfOH.

**Scheme 3:**
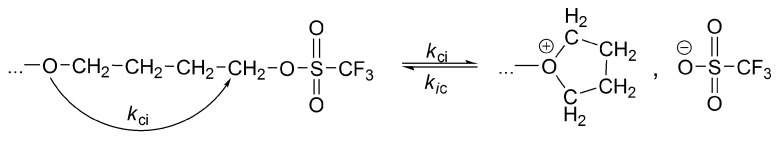
The intramolecular conversion of the covalent species (macroesters) into the ionic (macroion pairs) in the polymerization of THF initiated with TfOH.

**Scheme 4:**
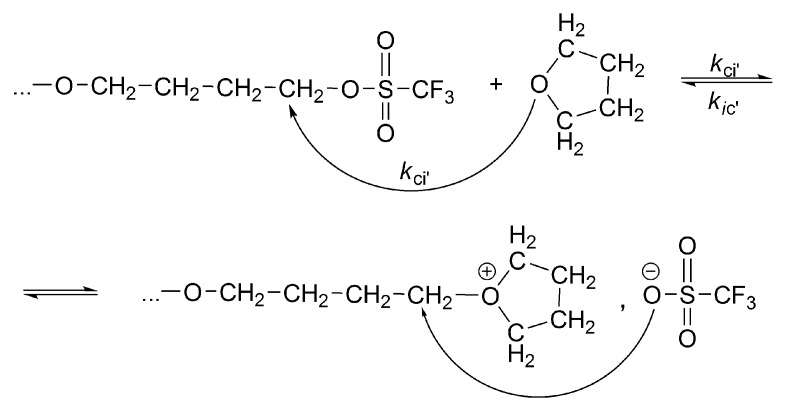
The intermolecular conversion of a macroester in reaction with a monomer molecule into an oxonium ion in the polymerization of THF initiated with TfOH.

**Scheme 5:**
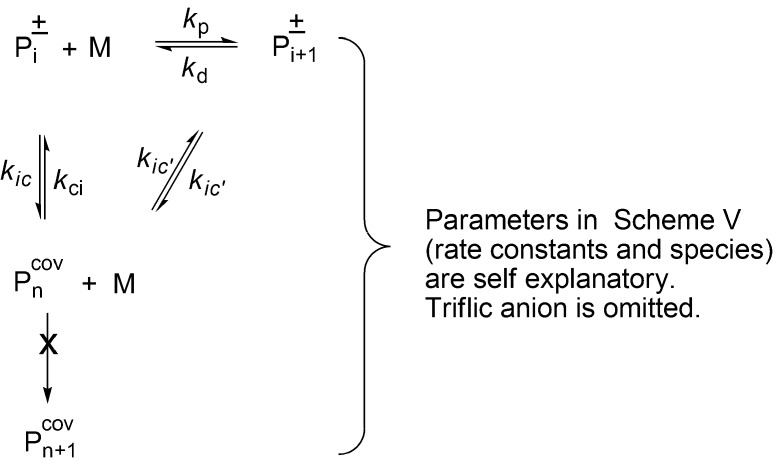
The set of ion–ester equilibria in the polymerization of THF initiated with TfOH.

The equilibrium involving active and dormant macromolecules that takes place in the ROP of THF initiated by TfOH is given in Scheme 2, Scheme 3, Scheme 4 and Scheme 5 [31].

**Figure 1:**
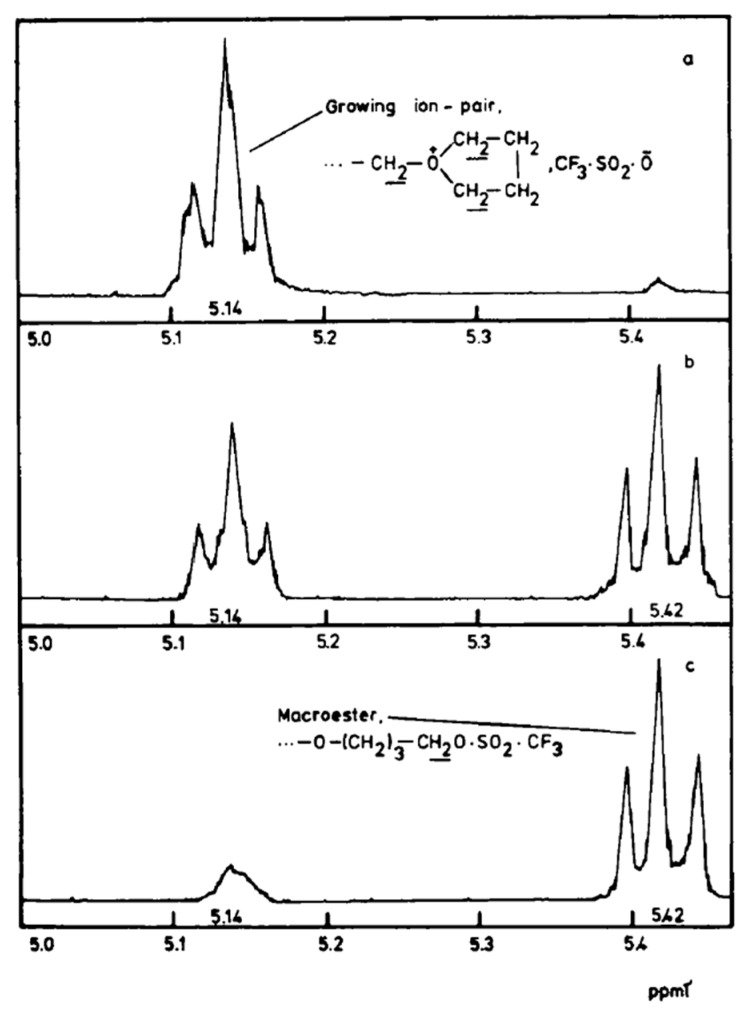
The 300-MHz ^1^H-NMR spectra (the 5.0–5.5 τ region only) of living polyTHF with CF_3_SO_2_O^−^ anion: (**a**) in CD_3_NO_2_; (**b**) in CH_2_Cl_2_; and (**c**) in CCl_4_ solvent (first published in [31], the scale is in τ units).

Due to the relatively slow ion-ester exchange rate (on the NMR time scale), both the covalent species and the ion pairs can be traced by ^1^H-NMR (Figure 1). Their relative concentrations strongly depend on the polarity of the solvent [31].

The conversion of the covalent species into the ionic ones proceeds with an anchimeric assistance (a neighboring group participation), and therefore is much faster than the reaction of the monomer with the covalent species (Scheme 3).

This is a unimolecular opposed reaction (an ion pair is kinetically one species), and since its rate is sufficiently low, its rate constant was measured by ^1^H-NMR [32]. It has been noticed that the direct covalent growth cannot take place, as it is forbidden by the symmetry rules. Covalent species may grow only by intermolecular conversion into ionic species, larger by one unit, when the macroester reacting with incoming monomer molecule is converted into an oxonium ion (as schematically shown in Scheme 4):

The complete set of equilibrating species in polymerization of THF initiated with triflic acid or its esters is schematically presented in Scheme 5 [33].

In the polymerization of THF initiated by triflic acid the rate constants for the various conditions have been measured. Since the detailed discussion of the mechanism of an ionic and a covalent propagation and determination of the rate constants is out of the scope of this paper, the interested reader may find these information in [31,32,33]. Nevertheless, establishing those kinetic parameters allowed determination the times of an average macromolecule to exist in ionic and covalent state. Furthermore, it could be calculated how many monomer molecules are added at these periods of time. These findings are illustrated below.

**Scheme 6:**
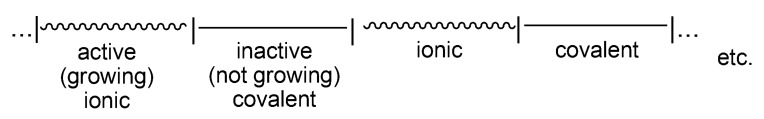
Periods of activity and inactivity (dormancy).

The record of life of a macromolecule that undergoes interconversions between active and dormant states can be visualized as sections marked on the line, where the lengths of these sections are equal to the time spent in a given state (Scheme 6). Thus, an average macromolecule propagates on ionic species for a certain time, adding X monomer molecules, then the ion-pair collapses into the covalent species. If these are sufficiently less reactive than the ionic species (*k*_p_ >> *k*_ci_), or unreactive at all, then the covalent species are dormant ones. Then, the macromolecule spends some time as an inactive (dormant) and recovers to the ionic state due to internal or external ionization (*k*_ci_ and/or *k*_ci’_).

**Scheme 7:**
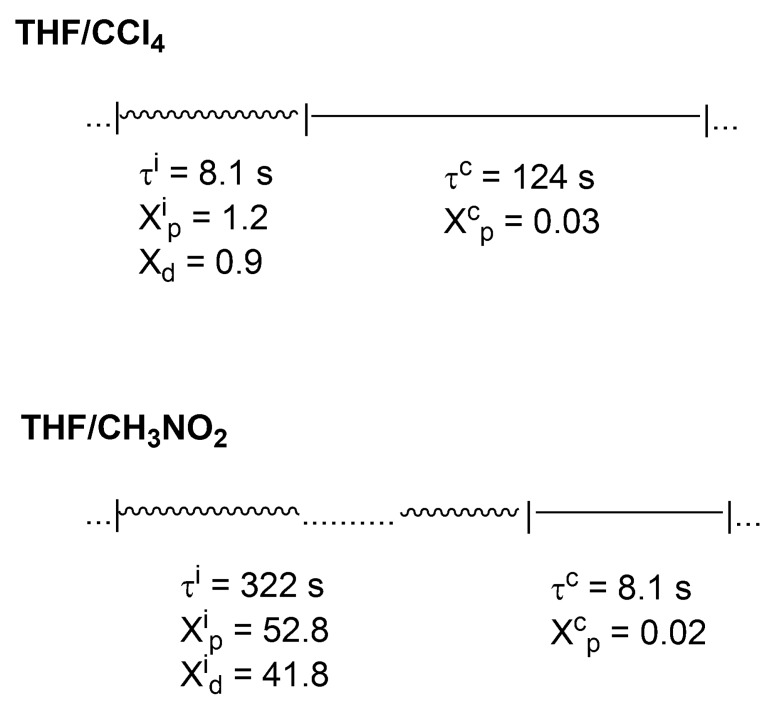
Conditions: [THF]_0_ = 8 mol·L^−1^; 25 °C [26,34], where τ is time, *X* is the number of monomer molecules, and indexes *i*, *c*, *p* and *d* stand for ionic and covalent propagation and depropagation, respectively.

In Scheme 7, it is shown how these active and inactive periods oscillate in the polymerization of THF in two solvents of different polarity (CCl_4_ and CH_3_NO_2_).

Thus, even in CCl_4_, where a macromolecule is in a dormant state much larger than in an ionic state (124 s vs. 8.1 s), there is approximately 10 times larger number of monomer molecules introduced into the chain by ionic chain ends. This ratio is even more than 500 in CH_3_NO_2_ solvent.

We have described the ionic polymerization of THF in some details, since it is the most general one (for ROP) and, moreover, the most thoroughly studied. Other polymerizations, e.g., polymerization of oxazolines or even the controlled radical polymerization, either proceed in the same way or are almost identical kinetically [34].

Polymerization of oxazolines (OX), with the interconversion between living and dormant species, has been elaborated in detail by Saegusa and Kobayashi [35,36]. More recently, kinetic analysis of these systems has been given by Dworak [37].

**Scheme 8:**
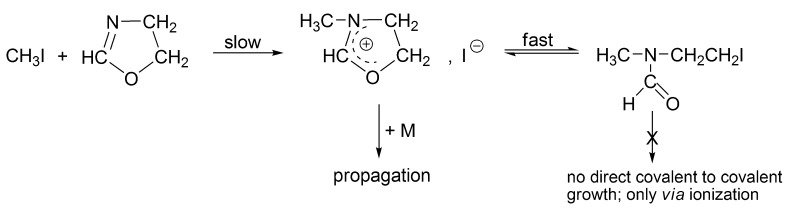
Polymerization of oxazoline with iodomethane [35,36].

The alkyl halides, in order to grow as such (covalent directly to covalent), must form the four-membered transition state, which is forbidden according to the symmetry rules. Thus, for the propagation to proceed, an interconversion of covalent-dormant species into the ionic ones is required. Ionization of covalent dormant species proceeds as for the polymerization of THF, almost exclusively intramolecularly (Scheme 8). Dworak has calculated, that intermolecular ionization with participation of monomer, in order to be competitive, would require the impossible to reach concentration of the monomer equal to ~10^3^ mol/L.

In all these systems, the interconversions are fast comparing with the rates of growth. Thus, there are several interconversions for one monomer molecule that reacted in an act of propagation [37].

## 5. Active–Dormant Interconversion in Polymerization of Cyclic Esters

The first catalytic system, successfully used in the polymerization of cyclic esters, was based on metal alkoxides (e.g., aluminum isopropoxide) and the polymerization could be considered as living and controlled. One of the first comprehensive papers discussing this issue has been published by the Liège group of Teyssié, Jerome and Dubois [38].

**Scheme 9:**
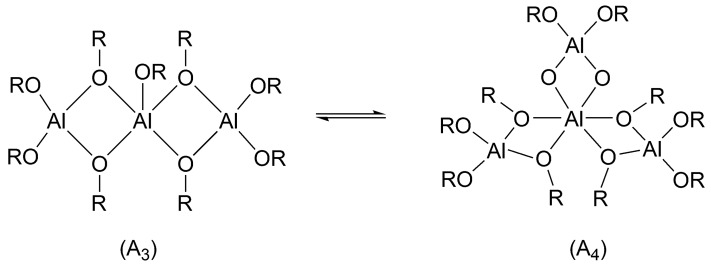
Trimer (A_3_) and tetramer (A_4_).

Aluminum isopropoxide exists in solutions as a mixture of a trimer and tetramer (Scheme 9).

There was a long-lasting controversy, whether all alkoxy groups initiate or only some of them. This controversy has been resolved in our institute, as we have shown in the polymerization of ε-caprolactone (CL) that in non-protonic solvents and at 25 °C in the mixture of a trimer and tetramer only trimer (cf. Scheme 9) initiates. When monomer conversion is complete, the tetramer remains intact [39]. On the other hand, Kricheldorf used higher temperatures (close to 100 °C) which caused all the alkoxy groups to be active (although the proportions of trimer and tetramer were not known) [40]. This means that at these conditions “dormant tetramer” has enough time to be converted into reactive trimer before polymerization is over. The difference in reactivities of trimer and tetramer stems from the fact, that in trimer the central Al atom is pentacoordinated and in tetramer hexacoordinated. Hexacoordinated atom does not have any coordination sites left to coordinate the monomer (cf. Scheme 9). Apparently, coordination with the central atom is a necessary condition for initiation. This may account, as Szwarc pointed out while discussing this system [25] (p. 123), for the thousand-fold faster reaction of trimer [39].

In many other systems, initiators and/or growing ionic macromolecules may form aggregates and only unimers are reactive or one of the forms of aggregates. Not knowing these subtle differences and not realizing the presence of inactive–dormant isomers, one may easily incorrectly determine concentration of active centers, and, as follows, the involved rate constants of elementary reactions.

**Scheme 10:**
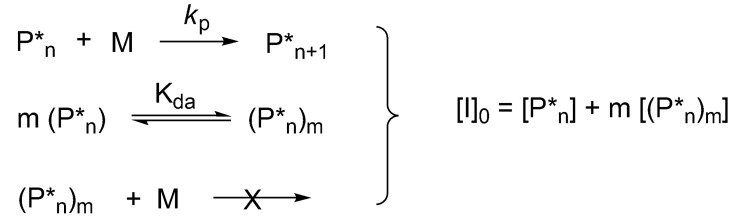
Kinetic system describing polymerization with dormant aggregated macromolecules and non-aggregated active macromolecules.

In the polymerization of cyclic esters formation of dormant aggregated macromolecules has been first shown for CL and *l*-lactide (LA), initiated by dialkylalkoxy aluminums, for which a kinetic equation has been developed. This allows simultaneous determination of the degree of aggregation “*m*”, equilibrium constant and rate constants of propagation [8]. In this case, the extent of aggregation can also be determined first from the dependence of the log of rate of polymerization, *r*_p_ on log [P*]_total_ ([P*]_total_ = [*I*]_0_), where [P*]_total_ is the total concentration of the active and dormant species—instantaneously active and dormant [41]. The corresponding kinetic system is shown in Scheme 10. The specific solution of equations describing this system for *m* = 2 has been given by Szwarc [25] (p. 108) and the general solution for any “*m*” has been proposed later [8].

If, based on Scheme 10, we assume that:

*m*[(*P*_n_^+^)_m_] >> [*P*_n_^+^], then m[(*P*_n_^+^)_m_] ≈ [*I*]_0_

As the rate of the polymerization equals:

*r*_p_ = −dln[*M*]/dt (1)

The combination of these two equations leads to (full derivation is given in [8]):

*r*_p_^1−*m*^ = −*m*·K_a_/*k*_p_*^m^*^−1^ + *k*_p_·[*I*]_0_·*r*_p_^−*m*^ (2)

Thus, “*m*” is known, and *k*_p_ and *K*_a_ can be determined from Equation (2) by plotting *r*_p_^1−*m*^ (the left hand side) as a function of [*I*]_0_·*r*_p_^−*m*^ (the second term of the right hand side of this equation).

**Figure 2:**
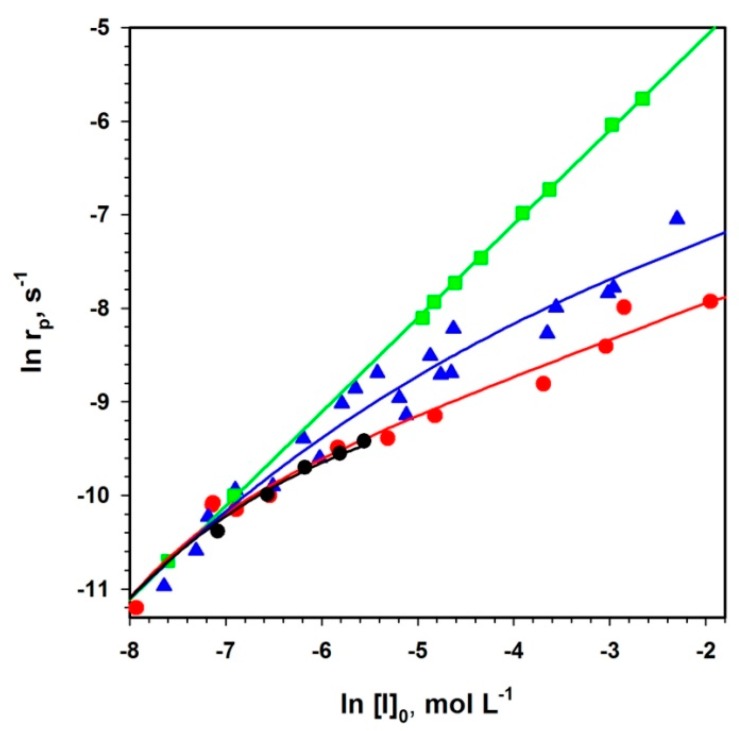
Bilogarithmic dependencies of the relative rates of polymerization (*r*_p_) of ε-caprolactone initiated with dialkyaluminum alkoxides on the starting concentrations of initiator ([*I*])_0_, showing influence of aggregation on the kinetics of the polymerization. Conditions: THF solvent, 25 °C, [ε-CL] = 2 mol·L^−1^. Points experimental for initiators: blue triangles (▲), (C_2_H_5_)_2_AlOC_2_H_5_; red circles (●), [(CH_3_)_2_CHCH_2_]_2_AlOCH_3_; black circles (●), (C_2_H_5_)_2_AlOCH_2_CH=CH_2_; green squares (■), (C_2_H_5_)_2_AlOCH_2_CH=CH_2_/(CH_3_)NCH_2_CH_2_NH(C_2_H_5_) (1/2 mole ratio). Experimental data have been taken from [41].

Plots showing the dependence of polymerization rate on the initial concentration of an initiator in the polymerization of ε-caprolactone (CL) with various R_2_AlOR’alcoholates are given in Figure 2.

Depending on the structure of active species and conditions of polymerization, propagation proceeds on the species that either retain their integrity independently of concentration (green squires) or partially aggregate into the temporarily inactive species as described by the Equation (2).

The ability to aggregate and the size of the aggregates depend on the entourage of the active species. Thus, for R_2_AlOR’ as an initiator, there is aggregation into trimer when R = C_2_H_5_, into a dimer for *i*-C_4_H_9_ and no aggregation when active species are solvated by amino ligands. The absence of aggregation for propagation on Al(*i*-OPr)_3_ (not shown) is particularly spectacular, since the initiator itself is highly aggregated (cf. above) and disaggregates upon propagation, when *i*-OPr groups on the Al atoms are converted into macromolecules.

**Figure 3:**
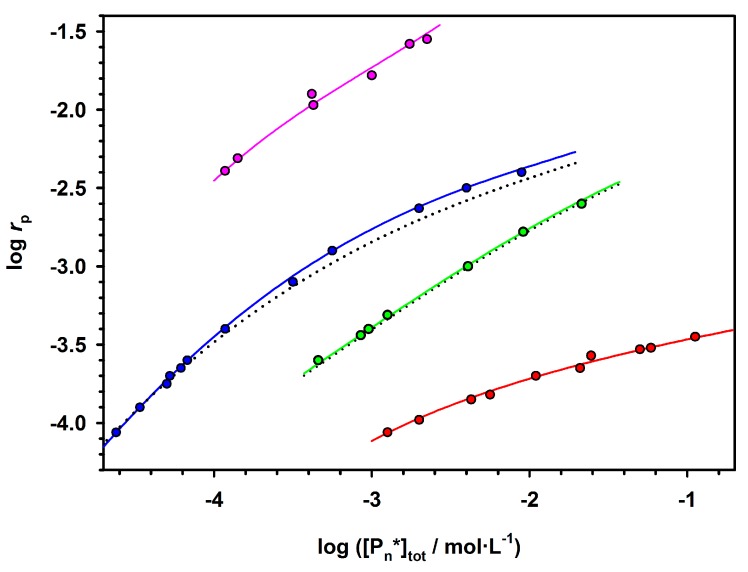
Green plot: The polymerization of 2-methoxystyrene with Li^+^ ion, in toluene as a solvent, at 20 °C (the experimental data taken from [42]). Pink: An anionic polymerization of methyl methacrylate with Li^+^ cation, in THF as a solvent, at −65 °C (the experimental data taken from [43]). Blue: The polymerization of oxirane on −CH_2_CH_2_O^−^, Cs^+^ ion pairs, in THF as a solvent, at 70 °C (the experimental data taken from [44]). Red: The polymerization of hexamethylcyclotrisiloxane (D_3_) on −Si(CH_3_)_2_O^−^, Li^+^ ion pairs, in THF as a solvent, at 22 °C (the experimental data taken from [45]). Graph has been taken from our paper [8].

The presented method (Equation (2)) of analysis of polymerizations with aggregation of active centers that leads to dormancy allowed analyzing the already published data not only for ROP but also for some vinyl polymerizations (Figure 3).

**Figure 4:**
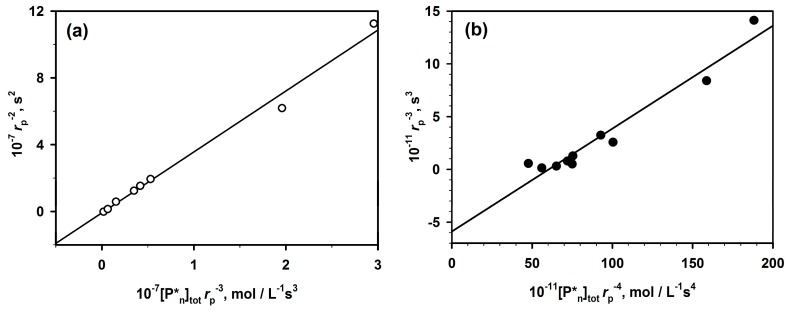
Polymerization of: oxirane (**a**); and hexamethylcyclotrisiloxane-D_3_ (**b**). Conditions are in the description of Figure 3. Graphs have been taken, as above, from [8].

In Figure 4, two chosen dependences from Figure 3 are analyzed according to Equation (2). The rates of polymerization (with 1-m exponent) are plotted against the total concentration of the active centers (equal to the starting concentration of an initiator multiplied by *r*_p_^−*m*^). The straight lines provide *k*_p_ from intercepts and *K*_a_ from the slopes. It is shown in the original paper that improper values of m do not give straight lines.

The determined in this way values of constants have been found practically equal to the values measured by the authors of the original papers who were using numerical method of fitting. It proves the utility of the linear analytical approach and the corresponding equation that gives an access to the analytical description of the polymerization with a reversible formation of dormant aggregates.

## 6. Dormancy in Sn(Oct)_2_/ROH Catalyzed/Initiated Polymerization of Cyclic Esters: l-Lactide (LA) and ε-Caprolactone (CL)

**Scheme 11:**
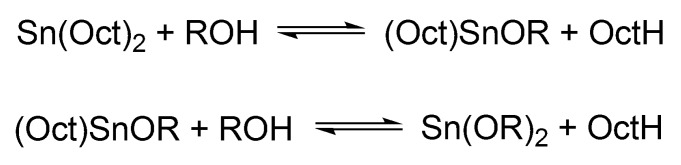
Sn(Oct)_2_, (Oct)SnOR, Sn(OR)_2_ equilibria.

Sn(Oct)_2_/ROH catalyzed/initiated polymerization of cyclic esters will be discussed separately for its importance in a commercial polymerization of LA in the melt and a wide application in research works on a cyclic esters polymerization (altogether over 1000 papers, according to the Web of Sci.). In polymerization of cyclic esters Sn(Oct)_2_ as such is not active. It gains the activity only after the reaction with, e.g., alcohols, when it is converted first into mono- and finally into dialkoxy tin (Sn(OR)_2_) (cf. Scheme 11).

**Scheme 12:**
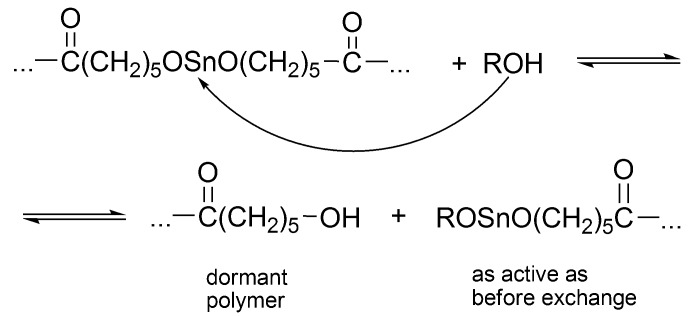
Chain transfer and creation of dormant species in the polymerization of ε-caprolactone with the Sn(Oct)_2_/ROH system.

Dialkoxy tin formally behaves as other metal alkoxides [38]. The alcohol added to the system acts both as an initiator and as a chain transfer agent, which reversibly forms dormant species until it becomes an end group of a macromolecule (Scheme 12).

**Figure 5:**
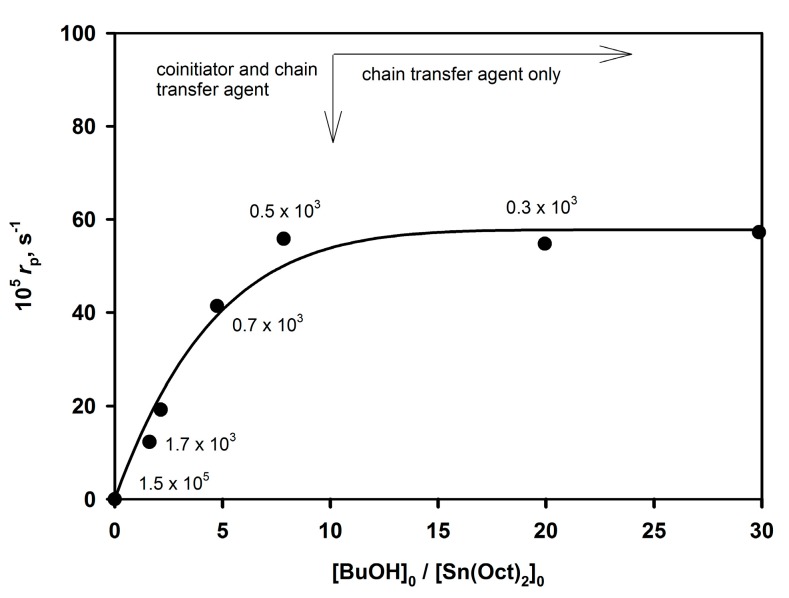
Dependence of the rate of polymerization of l-Lacide (LA) ((*r*_p_ = ln{([LA]_0_ − [LA]_eq_)/([LA] − [LA]_eq_)}/*t*) on [*n-*BuOH]_0_) at constant concentration of Sn(Oct)_2_ = 0.05 mol·L^−1^. Polymerization at 50 °C, in THF solvent. The numbers on the plot relate to *M*_n_ values [46].

The [ROH]_0_/[Sn(Oct)_2_]_0_ ratio first strongly influences the rate of the polymerization until it reaches plateau. The established relationship is illustrated in Figure 5.

Approximately tenfold excess of BuOH over Sn(Oct)_2_ is needed to convert all Sn(Oct)_2_ in the system into active species. When no BuOH is added, *M*_n_ of the obtained polymer approaches 10^5^ and apparently traces amounts of H_2_O or other proton donors present in the system serve as a coinitiator.

According to Figure 5, at first, the rate of the polymerization increases with increasing [*n*-BuOH] (the active species are formed according to Scheme 11) and then it reaches a plateau when the concentration of active sites becomes invariant. Since the number of active species is constant and *P*_n_ is equal to the [monomer]_0_/[BuOH]_0_ ratio at complete conversion, the system is both living and controlled.

Apparently, a similar (formally) mechanism takes place for the polymerization of LA initiated/catalyzed by a strong base (e.g., 1,8-diazabicyclo[5.4.0]undec-7-ene (DBU)) and an alcohol. When alcohol is absent, then as it has been shown by Waymouth, macrocyclic PLA are formed [47,48].

**Scheme 13:**
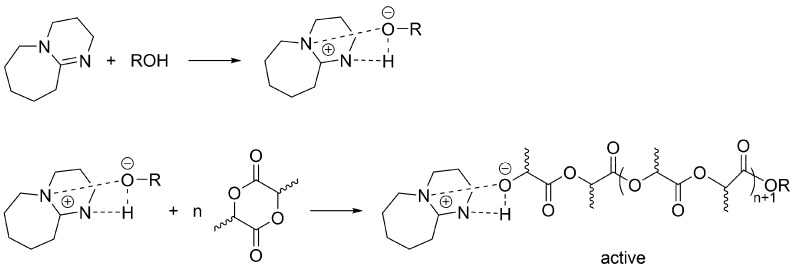
Polymerization of l-lactide with DBU/alcohol system. Charges (in place of partial charges) are schematically shown for clarity although the complete ionization and presence of ions have not yet been experimentally established.

Recently, a comprehensive study of the lactide LA polymerization with DBU/alcohol has been published [49]. This study encompasses the earlier works and shows, in agreement with previous observations, a simultaneous coexistence of an unreacted DBU, DBU H-bonded with growing macromolecules and dormant HO-ended macromolecules. Authors have shown that when sufficient excess of an alcohol over DBU is used, the process involves activated alcohol pathway, in which growing species are presented as activated –OH growing species (Scheme 13). However, since termination has been found, it is considered living and controlled only in a certain region of concentrations of monomer, DBU and an alcohol, as well as only at limited conversion and polymerization degree.

According to the postulated mechanism, the first order deactivation of the active macromolecules takes place, forming temporarily inactive, dormant, –OH ended macromolecules, accompanied with deactivation of DBU.

**Scheme 14:**
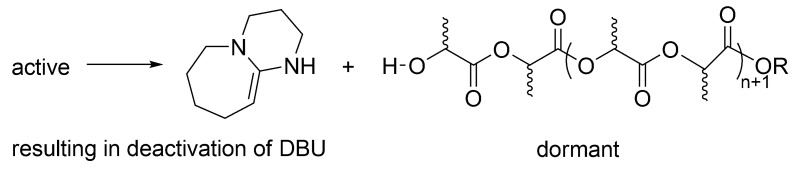
Active–dormant conversion in the polymerization of l-lactide with DBU/alcohol system.

Macromolecules with –OH end-groups are dormant, since can be reactivated by DBU (Scheme 14). This is, however, for as long as there is still DBU present, since it is deactivated as shown above. At that stage, when there is no more DBU, formation of the –OH ended macromolecules (Scheme 14) would be a termination reaction.

**Scheme 15:**
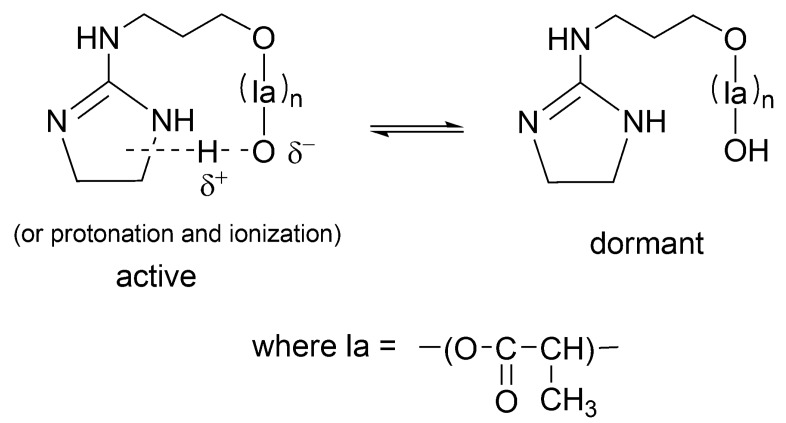
The proposed intramolecular mechanism of active–dormant interconversion for 3-[(4,5-dihydro-1*H*-imidazol-2-yl)amino]-propanol INICAT system (in [50] the intermolecular activation has also been discussed).

Related way of formation of dormant macromolecules has also been postulated for other strong base + alcohol systems [48] as well as for catalyst acting simultaneously as initiator (INICAT) [50]. In the latter system, we postulated the following equilibrium with formation of dormant species as shown in Scheme 15.

## 7. Formation of Dormant Species in Polymerizations of Vinyl Monomers

In this subsection, formation of dormant species in the active inactive 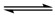 interconversion in vinyl polymerization will be briefly presented for comparison with ROP.

The best known example of such transformation is an anionic polymerization of dienes with Li^+^ counterion discussed in papers by Szwarc, Bywater and Fetters [25] (p. 34). The aggregation in this system gives a very good example of dormant species formation. However, discussion used to be so extensive and so well known that we will restrain ourselves from getting into any details.

**Scheme 16:**
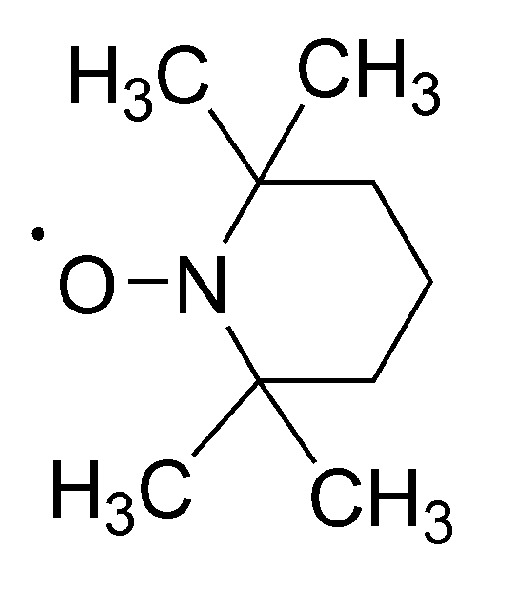
TEMPO—a stable radical.

**Scheme 17:**
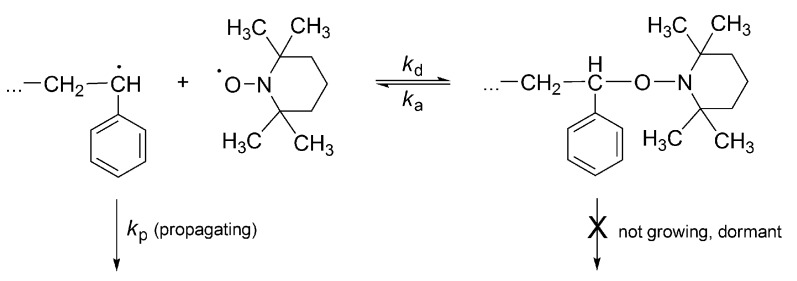
CRP of styrene with TEMPO [52].

No less spectacular is the “Controlled Radical Polymerization: CRP” of vinyl monomers. In CRP, stable (persistent) radicals are used that are able to react with growing macroradicals, forming dormant species, although are unable to initiate polymerization or to react between themselves. The inactivating agents are in the so called “nitroxide moderated polymerization” (mostly 2,2,6,6-tetramethylpiperidinyl-1-oxide (TEMPO)) itself and its numerous derivatives (Scheme 16) [51]. The creation of dormant macromolecules in CRP of styrene with TEMPO is presented in Scheme 17.

**Scheme 18:**
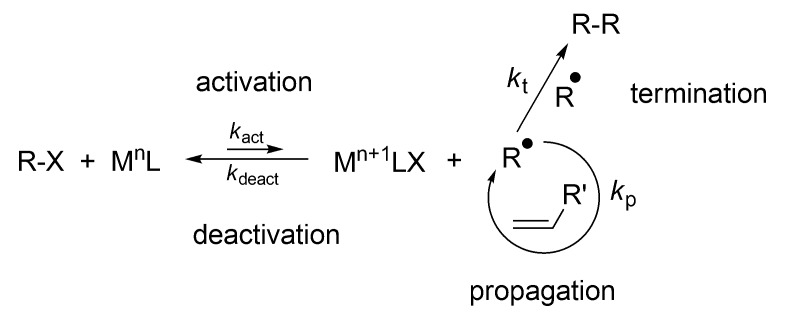
Schematic illustration of the key reactions in ATRP. (L stands for ligand—mostly derivatives of amines, and M is Cu on the oxidation state *n* or *n* + 1).

Even more widely used is the process known as atom transfer radical Polymerization (ATRP), discovered in 1995 by Matyjaszewski [11]. This process involves a reversible reaction of the macroradicals with Cu^II^ salts, that provides reversible radical complex which is neither able to initiate polymerization, nor to react with itself (like TEMPO), (Scheme 18).

The fast buildup of M^n+1^·LX (e.g., Cu^II^Cl_2_/ligand) establishes the persistent radical effect and controls termination [9,11]. However, in radical polymerizations at least some termination by combination or disproportionation of the macroradicals is inevitable. Nevertheless, conditions have been found allowing carrying the polymerization to the full monomer conversion with only a few percent of macromolecules terminated at the end of the process. To the same category belongs Reversible addition-fragmentation chain-transfer polymerization (RAFT), as described in Reference [52].

**Scheme 19:**
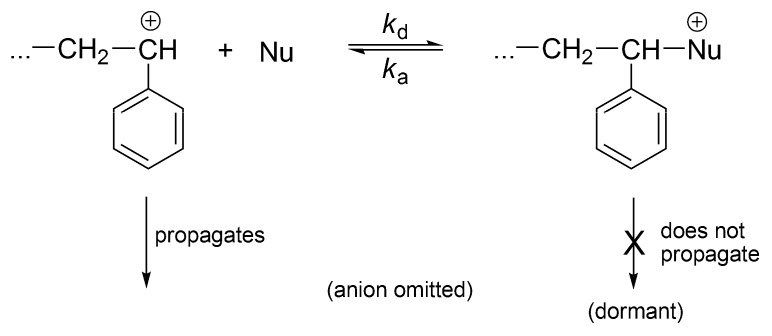
The cationic polymerization of styrene with added nucleophile.

The cationic polymerization can also be a controlled process if the nucleophiles are introduced into the system converting reversibly the instantaneously active into the dormant ones (Scheme 19).

This process, at certain conditions, is well controlled. It is not living, since carbocations easily undergo proton transfer (except at very low temperatures).

**Scheme 20:**
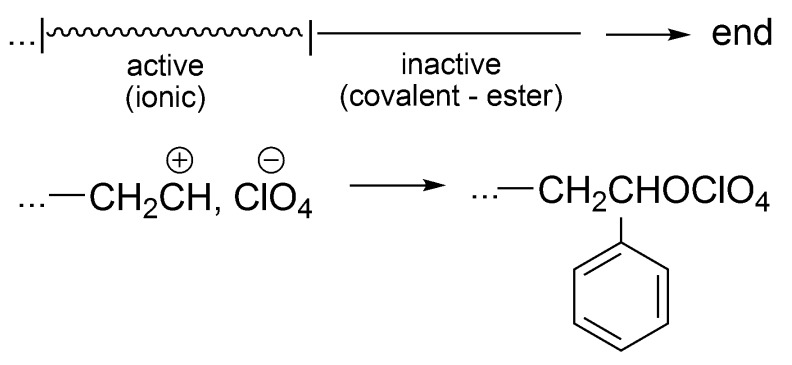
The polymerization of styrene with HClO_4_ to partial conversion only.

The cationic vinyl polymerization with added nucleophiles has been comprehensively described in terms of reversible formation of dormant species in the Sigwalt’s laboratory [53] (Scheme 20). Indeed, Sigwalt has measured the ratio *k*_p_/*k*_tr_ in systems with and without nucleophiles and have found the same values [54]. Thus, it has been clearly demonstrated that the only propagating species are usual carbocations and the other species, formed in reaction with nucleophiles, are just non-propagating, dormant macroions (oxonium, ammonium ions, etc.). These onium cations reversibly form back carbocations. Therefore, controlled polymerization, close to the living, results from formation of dormant macromolecules in the reaction of macrocations with nucleophiles. Polymerization becomes much slower and better conditions for the control of the process are provided.

In this way, the original idea, that addition of nucleophiles (ethers, esters, sulfides) forms a new kind of active (i.e., growing) species has finally been put to rest. Thus, let us quote Szwarc, who had written on this subject: “No new mechanism operates “living” cationic polymerization. Neither does a new kind of species participate in these polymerizations” [25] (p. 142). It has to be understood that onium ions are not propagating by themselves.

Formation of dormant species has also been shown in styrene cationic polymerization initiated by protonic acids. Even though the cationic polymerization of styrene initiated by perchloric acid (HClO_4_) does not have the importance of ATRP, it has to be at least briefly mentioned, as it has triggered several further works involving dormant species. It shows additionally that the same system, depending on conditions (e.g., temperature), is living, living/dormant or just with termination.

During the cationic polymerization of styrene initiated with HClO_4_, carried out at −97 °C, conversion of styrene stops at a certain conversion, that depends on the [styrene]_0_/[HClO_4_]_0_ ratio [55]. One molecule of acid produces at this temperature one macromolecule. This result is explained by the collapse of the living ion pairs and formation of the unreactive polystyrylperchloric ester (it cannot e.g., ionize back, transfer a proton and form any species able to restart polymerization at this temperature). Choosing proper [styrene]_0_/[HClO_4_]_0_ ratio polymerization may go to completion, showing then typical features of living/controlled process. Thus, in terms of our pictograms (as above for THF), there is one period of growth and, after collapse of counterions, the system is not active anymore. This process is a termination reaction (Scheme 20).

The perchloric ester at this temperature is not dormant, but just the termination product. If, however, the ratio [styrene]_0_/[HClO_4_]_0_ is properly chosen, then, polymerization may go to completion. At higher temperatures (e.g., −70 °C), the conversion of macroion into macroester is reversible and polymerization can be carried out to completion—the formed ester is a dormant species. Nevertheless, at still higher temperatures (e.g., at r.t.) the proton transfer takes place and up to 100 unsaturated macromolecules are formed from one acid molecule.

A similar system, namely with HOSO_2_CF_3_ as an initiator, has been discussed in details by Vairon, who has determined the involved lifetimes in the stop flow experiments [56]. At some conditions, there is a living polymerization with dormant species, at higher temperatures termination takes place.

## 8. Conclusions

The major common feature of the presented processes, both in ROP and in some vinyl polymerizations, is the fast interconversion between active and dormant species. Then, only a fraction of all macromolecules can propagate instantaneously. The total lifetime of an average macromolecule is much higher with an introduction of dormant species, as it is a sum of the time of activity and dormancy. The periods of activity and dormancy are characterized by the *k*_a_/*k*_d_ (rate constants of activation/deactivation) ratio that can be altered by experimentalist by choosing proper components of the system. An important result of the slowing down is the ability of all macromolecules to start polymerization at the same time leading to polymers of low dispersity if the rate of exchange growing 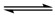 dormant is fast in comparison with the rate of propagation. In this way, polymerizations known for the long time as notoriously not living and without control are converted into systems with these desired features.

More examples of the formation of the dormant macromolecules in ROP and in vinyl polymerizations (e.g., in so-called “group transfer polymerization”) could be given, however the discussed examples are the most representative ones and the described phenomena also apply to other cases.

## References

[1] Szwarc M. (1968). Carbanions, Living Polymers and Electron Transfer Processes.

[2] Szwarc M., Jagur-Grodzinski J. (1974). Ions and Ion Pairs in Electron Transfer Reactions of Radical Anions, Carbanions and Solvated Electrons in Ions and Ion Pairs in Organic Reactions.

[3] Szwarc M. (1964). Review Lecture, Electron Transfer Reactions and Living Polymers. Proc. R. Soc..

[4] Szwarc M. (1998). Living Polymers. Their Discovery, Characterization, and Properties. J. Polym. Sci. Part A Polym. Chem..

[5] Grubbs R.R., Grubbs R.H. (2017). 50th Anniversary Perspective: Living Polymerization-Emphasizing the Molecule in Macromolecules. Macromolecules.

[6] Levy M. (2007). Impact of the Concept of “Living Polymers” on Material Science. Polym. Adv. Technol..

[7] Khanna S.N., Levy M., Szwarc M. (1962). Complexes Formed by Antracene with “Living” Polystyrene. “Dormant Polymers”. Trans. Faraday Soc..

[8] Duda A., Penczek S. (1994). Determination of the Absolute Propagation Rate Constant in Polymerization with Reversible Aggregation of Active Centres. Macromolecules.

[9] Greszta D., Mardare D., Matyjaszewski K. (1994). “Living” Radical Polymerization. 1. Possibilities and Limitations. Macromolecules.

[10] Veregin R.P.N., Georges M.K., Kazmaier P.M., Hamer G.K. (1993). Free Radical Polymerizations for Narrow Polydispersity Resins: Electron Spin Resonance Studies of the Kinetics and Mechanism. Macromolecules.

[11] Wang J.-S., Matyjaszewski K. (1995). Controlled/“Living” Radical Polymerization, Atom Transfer Radical Polymerization in the Presence of Transition Metal Complexes. J. Am. Chem. Soc..

[12] Fisher H. (1999). The Persistent Radical Effect in Controlled Radical Polymerization. J. Polym. Sci. Part A Polym. Chem..

[13] Szwarc M. (1956). “Living” Polymers. Nature.

[14] Szwarc M., Milkovich R., Levy M. (1956). Polymerization Initiated by Electron Transfer to Monomer. A new Method of Formation of Block Polymers. J. Am. Chem. Soc..

[15] Bodenstein M. (1913). Eine Theorie der photochemischen Reaktionsgeschwindigkeiten. Z. Phys. Chem..

[16] Penczek S., Pretula J.B., Matyjaszewski K., Moeller M. (2012). Fundamental Aspects of Chain Polymerization. Polymer Science: A Comprehensive Reference.

[17] Semenov N.N., translated by Frankel J.J. (1935). Chemical Kinetics and Chain Reactions.

[18] Hinshelwood C. (1964). Chemical Kinetics in the Past few Decades.

[19] Penczek S., Moad G. (2008). Glossary of Terms Related to Kinetics Thermodynamics, and Mechanisms of Polymerization (IUPAC Recommendation 2008). Pure Appl. Chem..

[20] Jenkins A.D., Kratochvíl P., Stepto R.F.T., Suter U.W. (1996). Glossary of Basic Terms in Polymer Science (IUPAC Recommendations 1996). Pure Appl. Chem..

[21] Szwarc M., van Beylen M. (1993). Ionic Polymerization and Living Polymers.

[22] Penczek S., Kubisa P., Szymanski R. (1991). On the Diagnostic Criteria of the Livingness of Polymerization. Macromol. Chem. Rapid Commun..

[23] Matyjaszewski K., Mueller A.H.E., Matyjaszewski K. (2009). Radical Polymerization. Controlled and Living Polymerizations.

[24] Inoue S. (2000). Immortal Polymerization: The Outset, Development and Application. J. Polym. Sci. Part A Polym. Chem..

[25] Szwarc M. (1996). Ionic Polymerization. Fundamentals.

[26] Penczek S., Szymanski R., Duda A. (1995). Polymerization with Contribution of Covalent and Ionic Species. Makromol. Symp..

[27] Flory P. (1940). Molecular Size Distribution in Ethylene Oxide Polymers. J. Am. Chem. Soc..

[28] Flory P. (1948). Fundamental Principles of Condensation Polymerization. Chem. Rev..

[29] Penczek S. (2000). Cationic Ring-Opening Polymerization (CROP) Major Mechanistic Phenomena. J. Polym. Sci. Part A Polym. Chem..

[30] Makina A., Kobayashi S. (2010). Chemistry of 2-oxazolines: A Crossing of Cationic Ring-Opening Polymerization and Enzymatic Ring-Opening Polymerization. J. Polym. Sci. Part A Polym. Chem..

[31] Matyjaszewski K., Penczek S. (1974). The Macroester 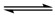 Macroion Equilibrium in the Cationic Polymerization of THF Observed Directly by 300 MHz ^1^H NMR. J. Polym. Sci. Polym. Chem. Ed..

[32] Buyle A.M., Matyjaszewski K., Penczek S. (1977). Kinetics and Thermodynamics of Interconversion of Macroesters and Macroion Pairs in Cationic Polymerization of Tetrahydrofuran. Macromolecules.

[33] Matyjaszewski K., Kubisa P., Penczek S. (1974). Ion 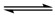 Ester Equilibrium in the Living Cationic Polymerization of Tetrahydrofuran. J. Polym. Sci. Polym. Chem. Ed..

[34] Penczek S., Kubisa P., Matyjaszewski K., Szymanski R., Goethals E.J. (1984). Structures and Reactivities in the Ring Opening and Vinyl Cationic Polymerization. Cationic Polymerizations and Related Processes.

[35] Saegusa T., Chujo Y. (1991). Macromolecular Engineering on the Basis of the Polymerization of 2-Oxazolines. Makromol. Chem. Macromol. Symp..

[36] Kobayashi S., Penczek S., Grubbs R.H. (2012). Polymerization of Oxazolines. Polymer Science: A Comprehensive Reference.

[37] Dworak A., Trzebicka B., Kowalczuk A., Tsvetanov C.H., Rangelov S. (2014). Polyoxazolines–Mechanism of Synthesis and Solution Properties. Polimery.

[38] Dubois P., Jerome R., Teyssie P. (1992). Macromolecular Engineering of Polylactones and Polylactides. 7. Structural Analysis of Copolymers of ε-Caprolactone and l- or d,l-Lactide initiated by Al(O^i^Pr)_3_. Macromolecules.

[39] Kowalski A., Duda A., Penczek S. (1998). Polymerization of l,l-Lactide Initiated by Aluminum Isopropoxide Trimer and Tetramer. Macromolecules.

[40] Kricheldorf H.R., Besl M., Schvarnagl N. (1988). Poly(lactones). 9. Polymerization Mechanism of Metal Alkoxide Initiated Polymerizations of Lactide and Various Lactones. Macromolecules.

[41] Duda A., Penczek S. (1994). Kinetics of Polymerization Involving Reversible Deactivation due to Aggregation of Active Centres—Analytical vs. Numerical—Solution for the Epsilon-Caprolactone Dialkylalkoxide Aluminum Systems. Macromol. Rapid Commun..

[42] Geerts J., van Beylen M., Smets G. (1969). Anionic polymerization of *o*- and *p*-methoxystyrene. J. Polym. Sci. Part A Polym. Chem..

[43] Kunkel D., Mueller A.H.E., Janata M., Lochman L. (1992). The role of association/complexation equilibria in the anionic polymerization of (meth)acrylates. Makromol. Chem. Macromol. Symp..

[44] Kazanskii K.S., Solovyanov A.A., Entelis S.G. (1971). Polymerization of ethylene oxide by alkali metal-naphthalene complexes in tetrahydrofuran. Eur. Polym. J..

[45] Wilczek L., Kennedy J.P. (1987). Aggregation in the Anionic Polymerization of Hexamethyl cyclotrisiloxane with Lithium Counterion. Polym. J..

[46] Kowalski A., Duda A., Penczek S. (2000). Kinetics and Mechanism of Cyclic Esters Polymerization Initiated with Tin(II)Octoate. 3. Polymerization of l,l-Dilactide. Macromolecules.

[47] Brown H.A., De Crisci A.G., Hedrick J.L., Waymouth R.M. (2012). Amidine-Mediated Zwitterionic Polymerization of Lactide. ACS Macro Lett..

[48] Lohmeijer B.G., Pralt R.C., Leibforth F., Logan J.W., Long D.A., Dove A.P., Nedesberg F., Choi J., Wade C., Waymouth R.M. (2006). Guanidine and Amidine Organocatalysts for Ring-Opening Polymerization of Cyclic Esters. Macromolecules.

[49] Scherck N.J., Kim H.Ch., Won Y-Y. (2016). Elucidating a Unified Mechanistic Scheme for the DBU—Catalyzed Ring-Opening Polymerization of Lactide to Poly(lactic acid). Macromolecules.

[50] Lewinski P., Sosnowski S., Penczek S. (2017). l-Lactide Polymerization—Living and Controlled—Catalyzed by Initiators: Hydroxy alkylated Organic Bases. Polymer.

[51] Jenkins A.D., Jones R.G., Moad G. (2010). Terminology for Reversible—Deactivation Radical Polymerization Previously Called “Controlled” Radical or “Living” Radical Polymerization. Pure Appl. Chem..

[52] Moad G., Solomon D.H. (2005). The Chemistry of Radical Polymerization.

[53] Matyjaszewski K., Sigwalt P. (1994). Unified Approach to Living and Nonliving Cationic Polymerization of Alkenes. Polym. Int..

[54] Sigwalt P., Poulton A., Tardi M. (1994). Transfer and Termination Reactions in Living Carbocationic Polymerization. J. Macromol. Sci..

[55] Darcy L.E., Millerine W.P., Pepper D.C. (1968). Molecular Weights in the Cationic Polymerization of Styrene by Perchloric Acid. Chem. Commun..

[56] Charleux B., Rives A., Vairon J.-P., Matyjaszewski K. (1998). Stopped Flow Investigations of Trifluoromethane Sulfonic Acid Initiated Cationic Oligomerization of Trans-1,3-diphenyl-1-butene. 2. A Model Kinetic Study of Styrene Cationic Polymerization. Macromolecules.

